# Immunomodulatory dietary polysaccharides: a systematic review of the literature

**DOI:** 10.1186/1475-2891-9-54

**Published:** 2010-11-18

**Authors:** Jane E Ramberg, Erika D Nelson, Robert A Sinnott

**Affiliations:** 1 Mannatech™, Incorporated, 600 S. Royal Lane, Suite 200, Coppell, TX 75019 USA

## Abstract

**Background:**

A large body of literature suggests that certain polysaccharides affect immune system function. Much of this literature, however, consists of *in vitro *studies or studies in which polysaccharides were injected. Their immunologic effects following oral administration is less clear. The purpose of this systematic review was to consolidate and evaluate the available data regarding the specific immunologic effects of dietary polysaccharides.

**Methods:**

Studies were identified by conducting PubMed and Google Scholar electronic searches and through reviews of polysaccharide article bibliographies. Only articles published in English were included in this review. Two researchers reviewed data on study design, control, sample size, results, and nature of outcome measures. Subsequent searches were conducted to gather information about polysaccharide safety, structure and composition, and disposition.

**Results:**

We found 62 publications reporting statistically significant effects of orally ingested glucans, pectins, heteroglycans, glucomannans, fucoidans, galactomannans, arabinogalactans and mixed polysaccharide products in rodents. Fifteen controlled human studies reported that oral glucans, arabinogalactans, heteroglycans, and fucoidans exerted significant effects. Although some studies investigated anti-inflammatory effects, most studies investigated the ability of oral polysaccharides to stimulate the immune system. These studies, as well as safety and toxicity studies, suggest that these polysaccharide products appear to be largely well-tolerated.

**Conclusions:**

Taken as a whole, the oral polysaccharide literature is highly heterogenous and is not sufficient to support broad product structure/function generalizations. Numerous dietary polysaccharides, particularly glucans, appear to elicit diverse immunomodulatory effects in numerous animal tissues, including the blood, GI tract and spleen. Glucan extracts from the *Trametes versicolor *mushroom improved survival and immune function in human RCTs of cancer patients; glucans, arabinogalactans and fucoidans elicited immunomodulatory effects in controlled studies of healthy adults and patients with canker sores and seasonal allergies. This review provides a foundation that can serve to guide future research on immune modulation by well-characterized polysaccharide compounds.

## Background

Polysaccharide-rich fungi and plants have been employed for centuries by cultures around the world for their dietary and medicinal benefits [1-5]. Often thought to merely support normal bowel function and blood glucose and lipid levels [6-8], certain polysaccharides have attracted growing scientific interest for their ability to exert marked effects on immune system function, inflammation and cancers [9-11]. Many of these chemically and structurally diverse, non- to poorly-digestible polysaccharides have been shown to beneficially affect one or more targeted cellular functions *in vitro *[11-16], but much of the *in vivo *literature consists of studies in which polysaccharides were injected [1,2]. For clinicians and scientists interested in immunologic effects following dietary intake, the value of such studies is uncertain. Polysaccharides that elicit effects *in vitro *or by injection may be ineffective or have different effects when taken orally [17]. We thus decided to conduct a systematic review to evaluate the specific immunologic effects of dietary polysaccharide products on rodents and human subjects.

## Methods

### Literature review

Studies were identified by conducting electronic searches of PubMed and Google Scholar from their inception to the end of October 2009. The reference lists of the selected articles were checked for additional studies that were not originally found in the search.

### Study selection and data extraction

The following search terms were combined with the term polysaccharide: dietary AND immune, or oral AND immune, or dietary AND inflammation, or oral AND inflammation. When specific polysaccharides or polysaccharide-rich plants and fungi were identified, further searches were conducted using their names with the same search terms. Studies were selected based on the following inclusion criteria:

1. Rodent or human studies

2. The presence of test group and control group (using either placebo, crossover, sham, or normal care)

3. Studies reporting statistically significant immunomodulatory effects

4. English language

5. Studies published up to October 2009.

Two researchers (JER, EDN) reviewed the list of unique articles for studies that fit the inclusion criteria. Uncertainties over study inclusion were discussed between the researchers and resolved through consensus. Searches were then conducted to obtain specific polysaccharide product information: safety (using the search terms: toxicity, NOAEL, LD_50_), composition and structure, and disposition.

### Quality assessment

Each study was assessed as to whether or not it reported a significant outcome measure for the polysaccharide intervention group.

## Results

A total of 62 rodent publications (Tables 1, 2 and 3) and 15 human publications (Table 4) were deemed appropriate for inclusion in this review. Available structural and compositional information for these immunomodulatory polysaccharides are provided in Table 5 and safety information is provided in Table 6. The majority of animal studies explored models in which animals were injected or implanted with cancer cells or tumors, were healthy, or were exposed to carcinogens. Other studies investigated immunodeficient, exercise-stressed, aged animals, or animals exposed to inflammatory agents, viruses, bacterial pathogens, pathogenic protozoa, radiation or mutagens. Human studies assessed immunomodulatory effects in healthy subjects, or patients with cancers, seasonal allergic rhinitis or aphthous stomatitis. Because of the limited number of human studies, we included some promising open-label controlled trials. Human study durations ranged from four days to seven years; daily doses ranging from 100-5,400 mg were reported to be well-tolerated.

**Table 1:** Immunomodulatory Glucan Extracts: Oral Animal Studies

Source	Extract	Animal	Dose/day	Duration of study	Treatment	Effects	Reference
*Agaricus* (*A. blazei*) *subrufescens*	α-1,6 and α-1,4 glucans	8-week ♀ C3H/He mice (5/group)	100 mg/kg IG every 3 days	1 month	Healthy animals	↑ #s splenic T lymphocytes (Thy1.2, CD4+ and CD8+)	[24]
			
	Aqueous	7-9-week ♂ Balb/cByJ mice (40/group)	1 ml 0.45N, 0.6N, or 3N aqueous extract	2 months		All doses ↑ serum IgG levels, CD3+ T cell populations and PML phagocytic activity	[22]
			
		7-9-week male Balb/cByJ mice (40/group)	1 ml 0.45N, 0.6N, or 3N aqueous extract	10 weeks	IP injection of OVA at 4 weeks	0.6N and 3N ↑ levels of OVA-specific serum IgG 28 days post-immunization; all doses ↑ delayed-type hypersensitivity and TNF-α secreted from splenocytes at 10 weeks; 0.6N ↑ splenocyte proliferation at 10 weeks	
		
		5-6 -week ♀ BALB/cHsdOla mice (8/group × 2)	One 200 μl extract day 1, orogastric intubation	1 week	Injected IP fecal solution day 2	↓ CFU in blood of mice with severe peritonitis & improved overall survival rate in all peritonitis groups	[46]
		
		6-week BALB/c nu/nu mice (7/group)	2.5 mg extract days 20-41, drinking water	41 days	Injected SC Sp-2 myeloma cells day 1	↓ tumor size & weight after 21 days treatment	[65]
	
	Aqueous, acid treated	6-week ♀ C57BL/6 mice (10/group)	20, 100 or 500 μg/ml, drinking water	9 days	Injected IP human ovarian cancer cells day 1	500 μg/ml ↓ tumor weight	[66]
				
			20, 100 or 500 μg/ml, drinking water	3 weeks	Injected IV murine lung cancer (3LL) cells	100 & 500 μg/ml ↓ #s metastatic tumors	
	
	Aqueous, with 200 ng/day β-glucan	6-week ♀ BALB/c mice (10/group)	200 ng days 5-21	3 weeks	Injected Meth A tumor cells day 1	↓ tumor size & weight	[23]
					
				2 weeks	Injected Meth A tumor cells	↑ cytotoxic T lymphocyte activity & spleen cell IFN-α protein	
				
			300 mg	5 days	Healthy animals	↑ splenic NK cell activity	

*Avena *spp.	β-glucans (particulate)	6-7 -week ♀ C57BL/6 mice (7/group)	3 mg every 48 h, days 1-3	1 month	Oral *E. vermiformis *oocytes day 10	↓ *E. vermiformis *fecal oocyte #s; increased intestinal anti-merozoite IgA; ↓ # of IL-4-secreting MLN cells	[42]
			
			3 mg on alternating days, days 1-10	22 days	Injected IP *Eimeria vermiformis *day 10	↓ *E. vermiformis *fecal oocyte #s; ↑ anti-merozoite intestinal IgA	[43]
	
	β-glucans (soluble)	4-week ♂ CD-1 mice (24/group)	0.6 mg/ml 68% β-glucan, drinking water	1 month	Resting or exercise-stressed (days 8-10) animals administered HSV-1 IN day 10	↓ morbidity in resting and exercise-stressed animals; ↓ mortality in exercise-stressed animals; pre-infection, ↑ Mø anti-viral resistance in resting and exercise-stressed animals	[38]
					
			~3.5 mg days 1-10, drinking water		Resting or exercise-stressed (days 5-10) animals administered HSV-1 IN day 10	Pre-infection, ↑ Mø antiviral resistance in resting animals	[41]
		
		4-week ♂ CD-1 mice (10/group)	0.6 mg/ml 68% β-glucan, drinking water	10 days	Resting animals or animals exposed to a bout of fatiguing exercise days 8-10 or moderate exercise days 5-10, injected IP with thioglycollate on day 10	↑ neutrophil mobilization in resting & moderately exercised animals; ↑ neutrophil respiratory burst activity in resting and fatiguing exercised animals	[37]
		
		4-week ♂ CD-1 mice (19-30/group)	0.8 mg/ml 50% β-glucan, days 1-10, drinking water	1 month	Resting or exercise-stressed (days 8-10) animals administered IN clodronate-filled liposomes to deplete Mø days 8 & 14 & infected IN with HSV-1 day 10	↓ morbidity, mortality, symptom severity in exercise-stressed animals, without Mø depletion	[40]
					
		4-week ♂ CD-1 mice (20/group)			Resting or exercise-stressed (days 8-10) animals administered HSV-1 IN day 10	↓ morbidity in exercise-stressed & resting animals; ↓ mortality in exercise-stressed animals	[39]

*Ganoderma lucidum*	Aqueous	7-week ♂ CD-1 mice (26/group)	5% of diet	5 months	Injected IM DMH once a week, weeks 1-10	↓ aberrant crypt foci per colon, tumor size, cell proliferation, nuclear staining of β-catenin	[69]
		
		4-8-week BALB/c mice (10/group)	50, 100 or 200 mg/kg, oral	10 days	Injected SD Sarcoma 180 cells	↓ of tumor weight was dose dependent: 27.7, 55.8, 66.7%, respectively	[67]

*Ganoderma lucidum *(mycelia)	Aqueous	7-week ♂ F344/Du Crj rats (16/group)	1.25% or 2.5% of diet	6 months	Injected SC AOM once a week, weeks2-5	Both doses ↓ colonic adenocarcinoma incidence; 2.5% ↓ total tumor incidence; both doses ↓ nuclear staining of β-catenin and cell proliferation	[68]

*Ganoderma tsugae*	Aqueous	8-week ♀ BALB/cByJNarl mice (14/group)	0.2-0.4% of diet (young fungi); 0.33 or 0.66% of diet (mature fungi)	5 weeks	Injected IP OVA days 7, 14, 21; aerosolized OVA twice during week 4	In splenocytes, both doses of both extracts ↑ IL-2 and IL-2/IL-4 ratios, 0.2% young extract and 0.66% mature extract ↓ IL-4; in Mø, 0.66% mature extract ↑ IL-1β, both doses of both extracts ↑ IL-6	[53]

*Grifola frondosa*	D fraction	Mice: 1) ICR, 2) C3H/HeN, 3) CDF_1 _(10/group)	1.5 mg every other day, beginning day 2	13 days	Implanted SC: 1) Sarcoma-180, 2) MM-46 carcinoma, or 3) IMC carcinoma cells	↓ tumor weight & tumor growth rate: 1) 58%, 2) 64%, and 3) 75%, respectively	[71]
		
		5-week ♂ BALB/c mice (10/group)	2 mg, days 15-30	45 days	Injected in the back with 3-MCA, day 1	↓ (62.5%) # of animals with tumors; ↑ H_2_0_2 _production by plasma Mø; ↑ cytotoxic T cell activity	[72]

*Hordeum vulgare*	β-1,3;1,4 or β-1,3;1,6-D-glucans	Athymic nu/nu mice (4-12/group)	40 or 400 μg IG for 4 weeks	31 weeks	Mice with human xenografts (SKMel28 melanoma, A431 epidermoid carcinoma, BT474 breast carcinoma, Daudi lymphoma, or LAN-1 neuroblastoma) ± mAb (R24, 528, Herceptin, Rituximab, or 3F8, respectively) therapy twice weekly	400 μg + mAb ↓ tumor growth & ↑ survival; higher MW ↓ tumor growth rate for both doses	[75]
	
	β-1,3;1,4-D-glucans	Athymic BALB/c mice	4, 40, or 400 μg for 3-4 weeks	1 month	Mice with neuroblastoma (NMB7, LAN-1, or SK-N-ER) xenografts, ± 3F8 mAb therapy twice weekly	40 and 400 μg doses + mAB ↓ tumor growth; 400 μg dose ↑ survival. Serum NK cells required for effects on tumor size	[76]
		
		C57BL/6 WT and CR3-deficient mice (10/group)	0.4 mg for 3 weeks	100 days	Injected SC RMA-S-MUC1 lymphoma cells day 1 ± IV 14.G2a or anti-MUC1 mAb every 3rd day	±mAB ↓ tumor diameter; ↑ survival	[73]
	
	β-glucans	♀ Fox Chase ICR immune-deficient (SCID) mice (9/group)	400 μg days 1-29	50 days	Mice with human (Daudi, EBV-BLCL, Hs445, or RPMI6666) lymphoma xenografts, ± Rituximab mAb therapy twice weekly	+mAB ↓ tumor growth and ↑ survival	[74]

*Laminaria digitata*	Laminarin	♂ ICR/HSD mice (3/group)	1 mg	1 day	Healthy animals	↑ Mø expression of Dectin-1 in GALT cells; ↑ TLR2 expression in Peyer's patch dendritic cells	[29]
		
		♂ Wistar rats (7/group)	5% of diet days 1-4, 10% of diet days 5-25	26 days	Injected IP *E. coli *LPS day 25	↓ liver ALT, AST, and LDH enzyme levels; ↑ ED2-positive cells, .↓ peroxidase-positive cells in liver; ↓ serum monocytes, TNF-α, PGE2, NO_2_	[44]

*Lentinula edodes*	SME	6-week nude mice	0.1 ml water with10% SME/10 g body weight days 1-19, 33-50	50 days	Injected SC prostate cancer (PC-3) cells day 1	↓ tumor size	[80]
	
	β-glucans	♀ 3- and 8-week BALB/c mice (15/group)	50, 100 or 250 μg	1-2 weeks	Healthy animals	250 μg dose ↑ spleen cell IL-2 secretion	[27]
			
		♀ 3- and 8-week BALB/c mice (15/group)	50, 100 or 250 μg	1-2 weeks	Injected murine mammary carcinoma (Ptas64) cells into mammary fat pads 2 weeks before treatment	↓ tumor weight	
	
	Lentinan	6-week ♂ Wistar-Imamichi specific-pathogen free rats (10/group)	1 mg twice weekly	1-2 months	Healthy animals	↑ T cell #s, helper-cell #s & helper/suppressor ratio, ↓ suppressor cell level at 4, but not 8 weeks	[26]
		
		5-6-week ♂ pre-leukemic AKR mice (10/group)	3 mg, days 1-7	3 weeks	Injected SC K36 murine lymphoma cells day 7	↓ tumor weight; ↑ tumor inhibition rate (94%)	[82]
					
		5-6-week athymic mice (10/group)		5 weeks	Injected SC colon cancer (LoVo and SW48, SW480 and SW620, or SW403 and SW1116) cells day 7	↓ tumor weight, ↑ tumor inhibition rate (>90%)	
		
		♂ AKR mice	3 mg	1 day	Pre-leukemic mice	↑ serum IFN-α and TNF-α, peak at 4 h and then back to normal at 24 h; ↑ IL-2 and IL-1α, peak at 2 h and back to normal at 24 h; ↑ CD3+ T, CD4+ T, CD8+ T, B lymphocytes	[81]

*Phellinus linteus*	Aqueous, alcohol-precipitated	6-7-week C57BL/6 mice (10-50/group)	200 mg/kg in drinking water	1 month	Healthy animals	↑ production and secretion of IFN-γ by con A stimulated T cells	[32]

*Saccharomyces cerevisiae*	Scleroglucan	♂ ICR/HSD mice (3/group)	1 mg one day before challenge (day 1)	6 days	IV *Staphylococcus aureus *or *Candida albicans *day 2	↑ long-term survival	[29]
	
	β-1,3;1,6 glucans (particulate)	3 and 8-week ♀ BALB/c mice (15/group)	50, 100 or 250 μg	1-2 weeks	Injected murine mammary carcinoma (Ptas64) cells into mammary fat pads 2 weeks before treatment	↓ tumor weight	[27]
						
	β-1,3-glucan				Healthy animals	All 3 doses ↑ phagocytic activity of blood monocytes & neutrophils & ↑ spleen cell IL-2 secretion	
		
		WT or CCD11b^-/- ^C57BL/6 mice (2/group)	0.4 mg for 3 weeks	100 days	Injected SC RMA-S-MUC1 lymphoma cells ± 14.G2a or anti-MUC1 mAb IV injection every 3^rd ^day	↓ tumor diameter when included with mAb; ↑ survival with and without mAb	[73]
		
		C57BL/6mice (4/group)	25 mg	1 week	Healthy animals	↑ # intestinal IELs; ↑ # TCRαβ+, TCR γδ+, CD8+, CD4+, CD8αα+, CD8αβ+ T cells in IELs; ↑ IFN-γ mRNA expression in IELs and spleen	[28]

*Sclerotinia sclerotiorum*	SSG	6-8-week specific pathogen-free ♂ CDF_1 _mice (3/group)	40 or 80 mg/kg days 1-10	2 weeks	Healthy animals	10 mg dose ↑ acid phosphatase activity of peritoneal Mø (day 14)	[30]
				
			40, 80 or 160 mg/kg days 2-6	35 days	Implanted SC Metha A fibrosarcoma cells day 1	80 mg dose ↓ tumor weight	
						
		6-8-week specific pathogen-free ♂ CDF_1 _mice (10/group)	40, 80 or 160 mg/kg days 2-11		Injected ID IMC carcinoma cells day 1		
		
		6-8-week specific-pathogen free ♂ mice of BDF1 and C57BL/6 mice (7/group)	0.5, 1, 2, or 4 mg days 1-10	2-3 weeks	Injected IV Lewis lung carcinoma (3LL) cells	2 mg ↓ # of 3LL surface lung nodules at 2 weeks	[83]

*Sclerotium rofsii*	Glucan phosphate	♂ ICR/HSD mice (3/group)	1 mg	1 day	Healthy animals	↑ systemic IL-6; ↑ Mø expression of Dectin-1 in GALT cells; ↑ TLR2 expression in dendritic cells from Peyer's patches	[29]

*Trametes *(*Coriolus*) *versicolor*	PSP	6-8-week ♂ BALB/c mice (10/group)	35 μg days 5-29 in drinking water	29 days	Implanted SC Sarcoma-180 cells day 1	↓ tumor growth & vascular density	[94]

**Table 2:** Immunomodulatory Non-Glucan Extracts: Oral Animal Studies

Extract	Source	Animal	Oral dose/day	Duration	Treatment	Significant effects	Reference
Fucoidans	*Cladosiphon okamuranus Tokida*	8-week ♀ BALB/c mice, 10/group	0.05% w/w of diet	56 days	DSS-induced UC	↓ disease activity index and myeloperoxidase activity; ↓ # of B220-positive colonic B cells; ↓ colonic MLN IFN-γ and IL-6 and ↑ IL-10 and TGF-β; ↓ colonic IgG; ↓ colonic epithelial cell IL-6, TNF-α, and TLR4 mRNA expression	[49]
	
	*Undaria pinnatifida*	5-week ♀ BALB/c mice (10-12/group)	5 mg, days 1-14 or 7-14	2 weeks	Injected HSV into cornea day 7	↓ facial herpetic lesions; ↑ survival, particularly in pre-treated animals	[45]
				
			10 mg	1 week	Administered 5-fluorouracil	↑ plasma NK cell activity	
						
					Injected SC HSV	↑ cytotoxic splenic T lymphocyte activity	
				
			0.1 or 0.5 mg	3 weeks	Injected IP HSV	Both doses ↑ serum neutralizing Ab titers, weeks 2 and 3	
		
		6-week ♂ ddY mice (5/group)	50, 100, 200 400 or 500 mg/kg days 1-28	3 weeks	Injected with Ehrlich carcinoma in back day 14	200-500 mg/kg ↓ tumor growth	[116]
			
		6-week ♂ BALB/c mice (8/group)	40 mg/kg alternating days 7-19	19 days	Injected IP Meth A fibrosarcoma day 1	↓ tumor growth	

Furanose (COLD-FX^®^)	*Panax quinquefolium*	Weanling ♂ SD rats (10/group)	450 or 900 mg/kg in food	1 week	Healthy animals	Both doses ↑ spleen Il-2 and IFN-γ production following ConA or LPS stimulation; ↓ proportion of total MLN and Peyer's patch CD3+ cells & activated T cells; high dose ↑ spleen cell IL-1β production following 48 h ConA stimulation.	[33]

Galacto-mannan (partially hydrolyzed guar gum)	*Cyamopsis tetragonolobus*	10-week ♀ BALB/c mice, 11-15/group	5% of diet	3 weeks	DSS-induced UC at beginning of week 3	↓ disease activity index scores, ↓ colonic mucosal myeloperoxidase activity & lipid peroxidation; ↓ colonic TNF-α protein levels & mRNA expression up regulated by DSS exposure	[50]
		
Galacto-mannans (guar gum)		8-month- SD rats, 5/group	5% of diet	3 weeks	Older animals	↓ serum IgG; ↑ MLN lymphocyte IgA, IgM and IgG production	[36]

Glucomannan (KS-2)	*Lentinula edodes*	DD1 mice (10-20/group)	140 mg/kg days 2-13	50 days	Injected IP Ehrlich ascites tumor cells day 1	↑ survival	[84]
				
			0.1, 1, 10, or 100 mg/kg dose days 2-13	100 days	Injected Sarcoma-180 tumor cells day 1	1, 10, and 100 mg/kg doses ↑ survival	

Heteroglycan (ATOM)	*A. subrufescens*	Mice (10/group): 1) 5-week ♂ Swiss/NIH; 6 week- ♀ DS mice; 3) 8-week ♀ BALB/c nude; 4) 5-week C3H/HcN	100 or 300 mg/kg days 2-11	8 weeks	Implanted SC 1) Sarcoma-180, 2) Shionogi carcinoma 42, 3) Meth A fibrosarcoma, or 4) Ehrlich ascites carcinoma cells	Both doses ↓ Sarcoma-180 tumor size at 4 weeks & ↑ survival; 300 mg/kg ↑ peritoneal macrophage and C3-positive cells; 300 mg/kg ↓ Shionogi and Meth A tumor sizes at 4 weeks. Both doses ↑ survival of Ehrlich ascites mice	[93]

Heteroglycan (LBP_3p_)	*Lycium barbarum*	♂ Kunming mice (10/group)	5, 10 or 20 mg/kg	10 days	Injected SC Sarcoma-180 cells	5 & 10 mg/kg ↑ thymus index; all doses ↓ weight, ↓ lipid peroxidation in serum, liver and spleen & ↑ spleen lymphocyte proliferation, cytotoxic T cell activity, IL-2 mRNA	[91]

Heteroglycan (PNPS-1)	*Pholiota nameko*	SD rats (5/group)	100, 200 or 400 mg/kg days 1-8	8 days	Implanted SC cotton pellets in scapular region day 1	↓ granuloma growth positively correlated with dose: 11%, 18% and 44%, respectively	[55]

Heteroglycan (PG101)	*Lentinus lepideus*	8-10-week ♀ BALB/c mice (3/group)	10 mg	24 days	6 Gy gamma irradiation	↑ colony forming cells, granulocyte CFUs/Mø, erythroid burst-forming units, and myeloid progenitor cells in bone marrow; induced proliferation of granulocyte progenitor cells in bone marrow; ↑ serum levels of GM-CSF, IL-6, IL-1β	[92]

Mixed poly-saccharides (Ambrotose^® ^or Advanced Ambrotose^® ^powders)	*Aloe barbadensis*, *Larix *spp, and other plant poly-saccharides	♂ SD rats (10/group)	37.7 or 377 mg/kg Ambrotose^® ^powder or 57.4 or 574 mg/kg Advanced Ambrotose^® ^powder	2 weeks	5% DSS in drinking water beginning day 6	574 mg/kg Advanced Ambrotose powder ↓ DAI scores; 377 mg/kg Ambrotose complex & both doses Advanced Ambrotose powder ↑ colon length and ↓ blood monocyte count	[52]

Pectin	*Pyrus pyrifolia*	6-8-week ♂ BALB/c mice (11/group)	100 μg days 1-7	22 days	Injected IP OVA day 7, provoked with OVA aerosol day 21	bronchial fluid:↓ IFN-γ & ↑ IL-5; splenic cells: ↑ IFN-γ, ↓ IL-5; normalized pulmonary histopathological changes; ↓ serum IgE	[54]

Pectins (bupleurum 2IIc)	*Bupleurum falcatum*	6-8-week ♀ specific-pathogen-free C3H/HeJ mice	250 mg/kg	1 week	Healthy animals	↑ spleen cell proliferation	[35]

Pectins (highly methoxylated)	*Malus *spp.	8-month- SD rats (5/group)	5% of diet vs. cellulose control	3 weeks	Older animals	↑ MLN lymphocyte IgA & IgG	[36]

Pectins	Citrus spp.	5-week ♀ F344 rats (30/group)	15% of diet	34 weeks	Injected SC AOM once a week, weeks 4-14	↓ colon tumor incidence	[86]
	
	*Malus *spp.	5-week ♀ BALB/c mice (6/group)	5% of diet	2 weeks	Healthy animals	↑ fecal IgA and MLN CD4+/CD8+ T lymphocyte ratio & IL-2 & IFN-γ secretion by ConA-stimulated MLN lymphocytes	[51]
			
		5-week ♀ BALB/c mice (6/group)	5% of diet days 5-19 vs. cellulose control	19 days	DSS-induced UC days 1-5	Significantly increased MLN lymphocytes IgA, and significantly decreased IgE; significantly decreased ConA-stimulated IL-4 and IL-10	
		
		4-week ♂ Donryu rats (20-21/group)	20% of diet	32 weeks	Injected SC AOM once a week, weeks 2-12	↓ colon tumor incidence	[85]
		
		4-week ♂ Donryu rats (19-20/group)	10 or 20% of diet	32 weeks	Injected SC AOM once a week, weeks 2-12	Both doses ↓ colon tumor incidence; 20% ↓ tumor occupied area & ↓ portal blood and distal colon PGE_2_	[90]

Pectins (modified)	Citrus spp.	2-4-month BALB/c mice (9-10/group)	0.8 or 1.6 mg/ml drinking water, days 8-20	20 days	Injected SC with 2 × 2 mm section of human colon-25 tumor on day 1	Both doses ↓ tumor size	[87]
		
		NCR nu/nu mice (10/group)	1% (w/v) drinking water	16 weeks	Orthotopically injected human breast carcinoma cells (MDA-MB-435) into mammary fat pad on day 7	↓ tumor growth rate & volume at 7 weeks, lung metastases at 15 weeks, # of blood vessels/tumor at 33 days post-injection	[89]
			
		NCR nu/nu mice (10/group)	1% (w/v) drinking water	7 weeks	Injected human colon carcinoma cells (LSLiL6) into cecum on day 7	↓ tumor weights and metastases to the lymph nodes and liver	
		
		SD rats (7-8/group)	0.01%, 0.1% or 1.0% wt/vol of drinking water, days 4-30	1 month	Injected SC MAT-LyLu rat prostate cancer cells	0.1% and 1.0% ↓ lung metastases; 1.0% ↓ lymph node disease incidence	[88]

**Table 3:** Immunomodulatory Polysaccharide-Rich Plant Powders: Oral Animal Studies

Source	Animal	Oral dose/day	Duration	Treatment	Significant effects	Reference
*Agaricus *(*A. blazei*) *subrufescens *(fruit bodies)	6-week ♂ C57BL/6, C3H/HeJ and BALB/c mice (3/group)	16, 32 or 64 mg	2 weeks	Healthy animals	32 and 64 mg ↑ liver mononuclear cell cytotoxicity	[25]

*Grifola frondosa*	6-week ♀ ICR mice (10-15/group)	5% of diet	36 weeks	Oral N-butyl-N'-butanolnitrosamine daily for first 8 weeks	↓ #s of animals with bladder tumors; ↓ tumor weight; ↑ peritoneal Mø chemotactic activity, splenic lymphocyte blastogenic response & cytotoxic activity	[70]

*Laminaria angustata*	Weanling SD rats (58/group)	5% of diet	26 weeks	IG DMBA, beginning of week 5	↑ time to tumor development and ↓ # of adenocarcinomas in adenocarcinoma-bearing animals	[77]

*Lentinula *(*Lentinus*) *edodes*	6-week ♀ ICR mice (10-17/group)	5% of diet	36 weeks	Oral BBN daily for first 8 weeks	↓ # of animals with bladder tumors; ↓ tumor weight; ↑ Mø chemotactic activity, splenic lymphocyte blastogenic response, cytotoxic activity	[70]
	
	7-8 -week ♂ Swiss mice (10/group)	1%, 5% or 10% of diet of 4 different lineages days 1-15	16 days	Injected IP N-ethyl-N-nitrosourea day 15	All 3 doses of one lineage and the 5% dose of two other lineages ↓ #s of micronucleated bone marrow polychromatic erythrocytes	[79]

*Lentinula edodes *(fruit bodies)	5-week ♀ ICR mice (14/group × 2)	10%, 20% or 30% of diet	25 days	Injected IP Sarcoma-180 ascites	All 3 doses ↓ Sarcoma-180 tumor weight	[78]
		
	Mice: 1) CDF_1_; 2) C3H; 3) BALB/c; 4,5) C57BL/6N (9/group × 3)	20% of diet	25 days	Injected SC 1) IMC carcinoma, 2) MM-46 carcinoma, 3) Meth-A fibrosarcoma, 4) B-16 melanoma, or 5) Lewis lung carcinoma cells	↓ growth of MM-46, B-16, Lewis lung, and IMC tumors; ↑ lifespan in Lewis lung and MM-46 animals	
		
	ICR mice (14/group × 2)	20% of diet days 1-7, days 7-31 or days 14-31	31 days	Injected IP Sarcoma-180 ascites	↓ tumor weight & growth when fed days 7-31 or 14-31	
		
	Mice: 1) CDF_1_; 2) C3 H (5/group × 4)	20% of diet	7-12 days	Injected SC: 1) IMC carcinoma or 2) MM-46 carcinoma cells	↑ spreading rate of activated Mø ↑ phagocytic activity	

*Phellinus linteus*	4-week ♂ ICR mice (10/group)	2 mg	1 month	Healthy animals	↓ serum & splenocyte IgE production; ↑ proportion of splenic CD4^+ ^T cells & splenocyte IFN-γ production	[31]

*Pleurotus ostreatus*	6-week ♀ ICR mice (10-20/group)	5% of diet	36 weeks	Oral BBN daily for first 8 weeks	↓ #s of animals with bladder tumors; ↓ tumor weight; ↑ plasma Mø chemotactic activity, splenic lymphocyte blastogenic response, cytotoxic activity	[70]

**Table 4:** Immunomodulatory Polysaccharide Products: Oral Human Studies

Extract	Source	Study design	Population	N (experimental/control)	Dose/day	Dura-tion	Significant effects	Reference
Arabino-galactans	*Larix occidentalis*	Randomized, double-blind, placebo-controlled	Healthy adults	8/15	4 g	6 weeks	↑ % CD8+ lymphocytes & blood lymphocyte proliferation	[18]
			
Arabino-galactans (ResistAid™)			Healthy adults given pneumococcal vaccinations day 30	21/24	4.5 g	72 days	↑ plasma IgG subtypes	[19]

Fucoidans	*Undaria pinnatifida *sporophylls	Randomized, single-blind, placebo-controlled	Healthy adults	25 (75% fucoidan, 6 (10% fucoidan)/6	3 g	12 days	75% fucoidan: ↓ #s blood leukocytes, lymphocytes' ↑ plasma stromal derived factor-1, IFN-γ, CD34+ cells; ↑ % CXCR4-expressing CD34+ cells	[21]

Furanose extract (Cold-FX^®^)	*Panax quinque-folium*	Randomized, double-blind, placebo-controlled	Healthy older adults given influenza immunization at the end of week 4	22/21	400 mg	4 months	During weeks 9-16, ↓ incidence of acute respiratory illness, symptom duration	[20]

Glucans	*Agaricus subru-fescens*	Randomized, double-blind, placebo-controlled	Cervical, ovarian or endometrial cancer patients receiving 3 chemotherapy cycles	39/61	5.4 g (estimated)	6 weeks	↑ NK cell activity, ↓ chemotherapy side effects	[64]

Glucans (β-1,3;1,6)	Not identified	Placebo-controlled	Recurrent aphthous stomatitis patients	31/42	20 mg	20 days	↑ PBL lymphocyte proliferation,↓ Ulcer Severity Scores	[48]

Glucans (β-1,3;1-6)	*S. cerevisiae*	Randomized, double-blind, placebo-controlled	Adults with seasonal allergic rhinitis	12/12	20 mg	12 weeks	30 minutes after nasal allergen provocation test, nasal lavage fluid: ↓ IL-4, IL-5, % eosinophils, ↑ IL-12	[47]

Glucans (PSK)	*Trametes versicolor*	Randomized, controlled	Patients with curatively resected colorectal cancer receiving chemotherapy	221/227	200 mg	3-5 years	↑ disease-free survival and overall survival	[56]
		
		Controlled	Post-surgical colon cancer patients receiving chemotherapy	123/121	3 g for 4 weeks, alternating with 10 4-week courses of chemo-therapy	7 years	↑ survival from cancer deaths; no difference in disease-free or overall survival	[57]
			
			Post-surgical colorectal cancer patients receiving chemotherapy	137/68	3 g daily	2 years	↑survival in stage III patients; ↓ recurrence in stage II & III patients	[58]
			
			Post-surgical gastric cancer patients receiving chemotherapy	124/129	3 g for 4 weeks, alternating with 10 4-week courses of chemo-therapy	5-7 years	↑ 5-year disease-free survival rate, overall 5-year survival	[59]
			
			Pre-surgical gastric or colorectal cancer patients	16 daily; 17 every other day/13	3 g daily or on alternate days before surgery	<14 days or 14-36 days	≥14 day treatment: ↑ peripheral blood NK cell activity, PBL cytotoxicity, proportion of PBL helper cells; ↓ proportion of PBL inducer cells; <14 day treatment: ↑ PBL response to PSK and Con A, proportion of regional node lymphocyte suppressor cells	[62]
		
		Randomized, double-blind, placebo-controlled	Post-surgical stage III-IV colorectal cancer patients	56/55	3 g for 2 months, 2 g for 22 months, 1 g thereafter	8-10 years	↑ remission & survival rates	[61]
		
		Controlled	Post-surgical stage III gastric cancer patients receiving chemotherapy	32/21	3 g	1 year	↑ survival time	[60]

Glucans (PSP)	*Trametes versicolor*	Randomized, double-blind, placebo-controlled	Conventionally-treated stage III-IV non-small cell lung cancer patients	34/34	3.06 g	1 month	↑ blood IgG & IgM, total leukocyte and neutrophil counts, % body fat; ↓ patient withdrawal due to disease progression	[63]

**Table 5:** Immunomodulatory Polysaccharide Products: Composition and Structure

Source	Category	Features	MW	Monosaccharide composition	Reference
*Agaricus subrufescens *(*A. blazei*)	Extract	β-1,6-D-glucan	10,000	NA	[66]

*Agaricus subrufescens *(fruit body)	Extract	α-1,6- and α-1,4 glucans with β-1,6-glucopyranosyl backbone (629.2 mcg/mg polysaccharides, 43.5 mcg/mg protein)	170,000	glucose	[24]
		
		α-1,4 glucans & β-1,6 glucans with β-1,3 side branches; α-1,6 glucans; β-1,6; 1-3 glucans, β-1,4 glucans; β-1,3 glucans; β-1,6; α-1,3 glucans; riboglucans, galactoglucomannans, β-1,2; β-1,3 glucomannans	NA	glucose, mannose, galactose, ribose	[25,117,118]

*Agaricus subrufescens *(mycelia)	Extract (ATOM)	β-1,6-D-glucan, protein complex, 5% protein	100,000-1,000,000	glucose, mannose, galactose, ribose	[93]

*Aloe barbadensis *(leaf gel)	Whole tissue	Dry weight: 10% polysaccharides; acemannan, aloemannan, aloeride, pectic acid, galactans, arabinans, glucomannans	average 2,000,000	mannose, glucose, galactose, arabinose, xylose, rhamnose	[119,120]
	
	Extract (aloemannan)	neutral partially acetylated glucomannan, mainly β-1,4-mannans	>200,000	mannose, glucose	[121]
	
	Extract (aloeride)	NA	4,000,000-7,000,000	37% glucose, 23.9% galactose, 19.5% mannose, 10.3% arabinose	[122]
	
	Extract (acemannan)	β-1,4 acetylated mannan	80,000	mannose	[123]

*Aloe barbadensis*, (leaf gel), *Larix *sp. (bark), *Anogeissus latifolia *(bark), *Astragalus gummifer *(stem), *Oryza sativa *(seed), *glucosamine*	Extracts (Ambrotose^® ^powder)	β-1,4 acetylated mannan, arabinogalactans, polysaccharide gums, rice starch, 5.4% protein	57.3% ≥ 950,000; 26.4% < 950,000 and ≥80,000; 16.3% ≤ 10,000	mannose, galactose, arabinose, glucose, galacturonic acid, rhamnose, xylose, fructose, fucose, glucosamine, galacturonic acid	(unpublished data, Mannatech Incorporated)
		
*Aloe barbadensis *(leaf gel), *Larix *sp. (bark), *Undaria pinnatifida *(frond), *Anogeissus latifolia *(bark), *Astragalus gummifer *(stem), *Oryza sativa *(seed), *glucosamine*	Extracts (Advanced Ambrotose^® ^powder)	β-1,4 acetylated mannan, arabinogalactans, polysaccharide gums, fucoidans, rice starch, 6% protein, 1% fatty acids	13% = 1,686,667; 46% = 960,000 30% <950,000 and ≥70,000; 11% ≤ 10,000		

*Avena *spp. (seed endosperm)	Extract	β-1,3;1,4 particulate (1-3 μ) glucans	1,100,000	glucose	[43]

*Avena *spp. (seed)	Extract	β-1,4,1,3 particulate glucans (linear chains of β-D-glycopyranosyl units; 70% β 1-4 linked)	2,000,000	NA	[41,124]

*Buplerum falcatum *(root)	Extract (bupleuran 2IIc)	6 linked galactosyl chains with terminal glucuronic acid substituted to β-galactosyl chains	NA	galactose, glucuronic acid, rhamnose	[35]

Citrus spp. (fruit)	Extract	α-1,4-linked partially esterified D-anhydrogalacturonic acid units interrupted periodically with 1,2-rhamnose	70,000-100,000	galactose, galacturonic acid, arabinose, glucose, xylose, rhamnose	[125]

*Cladosiphon okamuranus *(frond)	Extract	α-1,3-fucopyranose sulfate	56,000	fucose:glucuronic acid (6.1:1.0)	[126]

*Cordyceps sinensis *(mycelia)	Extract	β-1,3-D-glucan with 1,6-branched chains	NA	NA	[127]

*Cyamopsis tetragonolobus *(seed)	Extract (guar gum)	Main chain of β-1,4-mannopyranosyl units with α-galactopyranosyl units	220,000	mannose, galactose	[36,128]
	
	Extract (partially-hydrolyzed guar gum)	NA	20,000	mannose, galactose	[50]

*Flammulina velutipes*	Extract	NA	NA	glucose, mannose, galactose	[117]

*Flammulina velutipes *(fruit body)	Extract	β-1,3 glucan	NA	glucose	[129]

*Ganoderma lucidum*	Whole tissue	Linear β-1,3-glucans with varying degrees of D-glucopyranosyl branching, β-glucan/protein complexes, heteropolysaccharides	400,000-1,000,000	glucose, galactose, mannose, xylose, uronic acid	[130]
	
	Extract	NA	7,000-9,000	NA	[67]
	
*Ganoderma lucidum *(fruit body)	Extract	NA	7,000-9,000	NA	
		
		β-linked heteroglycan peptide	513,000	fructose, galactose, glucose, rhamnose, xylose (3.167: 0.556:6.89:0.549:3.61)	[15]

*Ganoderma tsugae*	Extract	55.6% carbohydrates (12.5% polysaccharides); 12% triterpenes, 1.7% sodium, 0.28% protein, 0% lipid	NA	NA	[53]

*Ginkgo biloba *(seed)	Extract	89.7% polysaccharides	NA	glucose, fructose, galactose, rhamnose	[131]

*Grifola frondosa*	Whole tissue	β-1,3; 1, 6-glucans, α-glucans, mannoxyloglucans, xyloglucans, mannogalactofucans	NA	glucose, fucose, xylose, mannose, galactose	[117]

*Grifola frondosa *(fruit body)	Extract (D fraction)	β-1,6-glucan with β-1,3 branches, 30% protein	NA	glucose	[132]
		
	Extract (X fraction)	β-1,6-D-glucan with α-1,4 branches, 35% protein	550,000-558,000	glucose	

*Hordeum *spp. (seed)	Extract	β-1,3;1,4-and β-1,3;1,6-D-glucans	45,000-404,000	glucose	[75]
		
		Primarily linear β-1,3;1,4- glucans	NA	glucose	[124]

*Laminaria *spp. (frond)	Extract (laminarin)	β-1,3;1-6 glucan	7,700	glucose	[29]
		
		β-1,3 glucan with some β-1,6 branches and a small amount of protein	4,500-5,500	glucose	[44]
	
	Extract	Fucoidan	NA	NA	[133]

*Larix occidentalis *(bark)	Extract	β-1,3;1,6-D-galactans with arabinofuranosyl and arabinopyranosyl side chains	19,000-40,000	galactose:arabinose (6:1), uronic acid	[128,134]

*Lentinula edodes*	Extract (SME)	β-1,3-glucans (4-5%), α-1,4-glucan (8-10%), protein (11-14%)	NA	glucose	[80]
	
	Extract	β-glucan	1,000	glucose	[27]
	
	Whole tissue	Linear β-1,3-glucans, β-1,4;1,6-glucans, heterogalactan	NA	glucose, galactose, mannose, fucose, xylose	[135]
	
	Extract (lentinan)	β-1,3-glucan with 2 β-1,6 glucopyranoside branchings for every 5 β-1,3-glucopyranoside linear linkages	500,000	glucose	[136]

*Lentinula edodes *(fruit body) *Lentinula edodes*	Extract (lentinan)	Neutral β-1,3-D glucan with two β-1,6 glucoside branches for every five β-1,3 units	400,000-800,000	glucose	[137]
		
	Extract (KS-2)	Peptide units and mannan connected by α-glycosidic bonds	60,000-90,000	mannose, glucose	

*Lentinula edodes *(mycelia or fruit body)	Extract	Triple helical β-1,3-D glucan with β-1,6 glucoside branches	1,000,000	glucose	[3]

*Lentinula edodes *(mycelia)	Extract (LEM)	44% sugars, 24.6% protein	~1,000,000	xylose, arabinose, glucose, galactose, mannose, fructose	[3]
	
	Extract (PG101)	72.4% polysaccharides, 26.2% protein, 1.4% hexosamine	NA	55.6% glucose, 25.9% galactose, 18.5% mannose	[138]

*Lycium barbarum*	Whole tissue	α-1,4;1,6-D-glucans, lentinan, β-1,3;1,6 heteroglucans, heterogalactans, heteromannans, xyloglucans	NA	glucose, galactose, mannose, xylose	[139]

*Lycium barbarum *(fruit body)	Extract (LBP_3p_)	88.36% sugars, 7.63% protein	157,000	galactose, glucose, rhamnose, arabinose, mannose, xylose (molar ratio of 1:2.12:1.25:1.10:1.95:1.76)	[91]

*Panax quinquefolium *(root)	Extract	Poly-furanosyl-pyranosyl saccharides	NA	arabinose, galactose, rhamnose, galacturonic acid, glucuronic acid	[33]
		
		NA	NA	glucose, mannose, xylose	[140]
	
	Extract (Cold-fX^®^)	90% poly-furanosyl-pyranosyl-saccharides	NA	furanose	[20]

*Phellinus linteus *(fruit body)	Extract	α- and β-linked 1,3 acidic proteoglycan with 1,6 branches	150,000	glucose, mannose, arabinose, xylose	[141]

*Phellinus linteus *(mycelia)	Extract	83.2% polysaccharide (4.4% β-glucan), 6.4% protein, 0.1% fat	NA	glucose	[142]

*Pholiota nameko *(fruit body)	Extract (PNPS-1)	NA	114,000	mannose, glucose, galactose, arabinose, xylose (molar ratio of 1:8.4:13.6:29.6:6.2)	[55]

*Pleurotus ostreatus *(mycelia)	Extract	β-1,3;1,6-D-glucans	316,260	glucose	[143]

*Saccharomyces cerevisiae*	Extract (WGP)	Particulate β-1,3;1,6-D-glucan	NA	glucose	[144]
	
	Extract	β-glucans with β-1,6 branches with a β-1,3 regions	NA	glucose	[124]
	
	Extract (SBG)	soluble β-1,3-D-glucan with β-1,3 side chains attached with β-1,6 linkages	20,000	glucose	[145]

*Sclerotinia sclerotiorum *(mycelia)	Extract (SSG)	β-1,3-D-glucan, <1% protein (>98% polysaccharide)	NA	glucose	[83]

*Sclerotium rofsii*	Extract (scleroglucan)	β-1,3;1,6 glucan	1,000,000	glucose	[29]

*Trametes versicolor *(fruit body)	Extract (PSP)	α-1,4, β-1,3 glucans, 10% peptides	100,000	glucose, arabinose, mannose, rhamnose	[146]

*Trametes versicolor *(mycelia)	Extract (PSK)	β-1,4;1,3;1,6-D-glucans, protein	94,000	glucose (74.6%), mannose (15.5%), xylose (4.8%), galactose (2.7%), fucose (2.4%)	[137,147]

*Undaria pinnatifida *(sporophyll)	Extract	Galactofucan sulfate	9,000	fucose:galactose 1.0:1.1	[148]
		
		Galactofucan sulfate	63,000	fucose:galactose:gluc-uronic acid (1.0:1.0:0.04)	[149]
		
		β-1,3-galactofucan sulphate	38,000	fucose, galactose	[150]

Unidentified source	Extract (modified citrus pectin)	NA	10,000	galactose, rhamnose, uronic acid	[125]
	
	Extract (highly methoxylated pectin)	NA	200,000	NA	[36]

**Table 6:** Safety of Immunomodulatory Polysaccharide Products Following Oral Intake

Category	Source	Test group	Test	Design	Results	Equivalent human dose*	Reference
Arabino-galactans	*Argemone mexicana *(arabinogalactan protein)	Pregnant rats	Develop-mental toxicity	250, 500, or 1,00 mg/kg, gestational days 5-19	No developmental toxicity: NOAEL = 1 g/kg	68 g	[151]
				
		♀ and ♂ rats	Fertility	250, 500, or 1,00 mg/kg, 1 month	No effects on reproduction: NOAEL = 1 g/kg		

Fucoidans	*Undaria pinnatifi*da	Rats	Subchronic toxicity	1.35 g/kg, 1 month	No evidence of toxicity	91.8 g	[152]

Galacto-mannans	*Cyamopsis tetragonolobus*	Adolescent and adult ♂ rats	Subchronic and chronic toxicity	8% of diet, 6-67 weeks	No evidence of toxicity	8% of diet	[153]
		
		Rats	Acute toxicity	One 7.06 g/kg dose: observed 2 weeks	LD_50 _= 7.06 g/kg	480 g	[96]
				
			Subchronic and chronic toxicity	1, 2, 4, 7.5 or 15% of diet, 3 months	All doses ↓ ♀ BW; 7.5-15% ↓ ♂ BW; 15% ↓ bone marrow cellularity; ↓ kidney and liver weights	1-15% of diet	
				
		19 adults with hypercholesterol-emia		18 g/day, 1 year	Short-term gastric bloating/loose stools, in 8 subjects, resolved in 7-10 days; 2 withdrew because of diarrhea. No toxicity for 13 subjects completing study	18 g	[154]
				
		16 Type II diabetics		26.4-39.6 g/day, 6 months	No effects on hematologic, hepatic, or renal function	39.9 g	[155]
							
		18 Type II diabetics		30 g/day, 4 months		30 g	
	
	*Cyamopsis tetragonolobus *(partially hydrolyzed guar gum)	Mice & rats	Acute toxicity	One 6 g/kg dose; observed 2 weeks	LD_50 _> 6 g/kg	>408 g	[156]
		
		Rats	Subchronic toxicity	0.2, 1.0 or 5% of diet, 13 weeks	No evidence of toxicity	5% of diet	
				
				0.5 or 2.5 g/kg, 1 month	NOAEL > 2.5 g/kg	>170 g	[157]
			
		*S. typhimurium*	Mutagenicity	Ames test	Not mutagenic	NA	

Glucans	*Agaricus subrufescens *(aqueous extract)	Rats	Subchronic toxicity	0.63, 1.25, 2.5 or 5% of diet, 3 months	NOAEL = 5% of diet	5% of diet	[158]
		
		3 women with advanced cancers	Case reports	Specific identity of products, doses, and durations of intake unknown	Severe hepatotoxicity; two patients died	NA	[97]
	
	*Agaricus subrufescens *(freeze dried powder)	24 normal adults and 24 adults with liver problems	Subchronic toxicity	3 g, 4 months	No evidence of toxicity	3 g	[159]
	
	*Ganoderma lucidum* (supplement)	Elderly woman	Case report	1 year *G. lucidum *(and another unidentified product, initiated one month previous)	Elevated liver enzymes and liver tissue damage	NA	[98]
	
	*Grifola frondosa *(powder)	Rats	Acute toxicity	One 2 g/kg dose	No evidence of toxicity	136 g	[160]
	
	*Lentinula edodes *(powder)	10 adults	Safety	4 g/day for 10 weeks; repeated 3-6 months later	50% of subjects experienced blood eosinophilia, ↑ eosinophil granule proteins in serum and stool, ↑GI symptoms	4 g	[99]
	
	*Lentinula edodes* (SME)	Nude mice	Safety	10% of diet days 1-18, 33-50	No adverse events	10% of diet	[80]
					
		61 men with prostate cancer		0.1 g/kg, 6 months	No adverse events	6.8 g	
	
	*Lentinus lepideus *(PG101)	Female mice	Subchronic toxicity	0.5 g/kg, 24 days	No evidence of toxicity	34 g	[92]
	
	*Phellinus linteus* (crude extract)	Rats	Acute toxicity	One 5 g/kg dose; observed 2 weeks	LD_50 _> 5 g/kg	349 g	[161]
	
	*Pleurotus ostreatus *(aqueous extract)	Mice	Acute toxicity	One 3 g/kg dose; observed 1 day	LD_50 _> 3 g/kg	>204.g	[100]
				
			Subacute toxicity	319 mg/kg, 1 month	Hemorrhages in intestine, liver, lung, kidney; inflammation and microabscesses in liver	21.7 g	
	
	*Saccharomyces cerevisiae *(particulate glucan [WGP])	Rats	Acute toxicity	One 2 g/kg, observed 2 weeks	LD_50 _> 2 g/kg	>136 g	[144]
				
			Subchronic toxicity	2, 33.3 or 100 mg/kg, 3 months	NOAEL = 100 mg/kg	6.80 g	

Heteroglycans	*Trametes versicolor* (PSP)	Rats	Subchronic toxicity	1.5, 3.0 or 6.0 mg/kg, 2 months	No evidence of toxicity	408 mg	[162]
			
		Rats & monkeys	Subchronic and chronic toxicity	100-200X equivalent human dose, 6 months	No evidence of toxicity	NA	
	
	*Trametes versicolor* (PSK)	Humans with colon cancer	Safety	3 g/day, up to 7 years	No significant adverse events	3 g	[57]
						
		Humans with colorectal cancer		3 g/day, 2 years		3 g	[58]

Mannans	*Aloe vera *gel	Dogs	Acute toxicity	Fed one 32 g/kg; observed 2 weeks	LD50 > 32 g/kg	>2,176 g	Bill Pine, personal communi-cation
					
		Rats		One 21.5 g/kg; observed 2 weeks	LD50 > 10 g/kg	>680 g	

*150 lb adult

A number of studies in healthy human adults demonstrated immune stimulating effects of oral polysaccharides. Arabinogalactans from *Larix occidentalis *(Western larch) were shown in RCTs to increase lymphocyte proliferation and the number of CD8+ lymphocytes [18] and to increase the IgG subtype response to pneumococcal vaccination [19]. A furanose extract from *Panax quiquefolium *(North American ginseng) was shown in an RCT of healthy older adults to decrease the incidence of acute respiratory illness and symptom duration [20]. Finally, an RCT of healthy adults consuming *Undaria pinnatifida *(wakame) fucoidans found both immune stimulating and suppressing effects, including increased stromal-derived factor-1, IFN-g, CD34+ cells and CXCR4-expressing CD34+ cells and decreased blood leukocytes and lymphocytes [21].

Studies in healthy animals showed a number of immune stimulating effects of various glucan products from *Agaricus subrufescens (A. blazei) *(aqueous extracts [22], aqueous extracts with standardized β-glucans [23], α-1,6 and α-1,4 glucans [24], and whole plant powders [25]); *Lentinula edodes *(shiitake) (lentinan [26] and β-glucans [27]); *Saccharomyces cerevisiae *(β-1,3-glucans [27,28]); *Laminaria digitata *(laminarin [29]); *Sclerotium rofsii *(glucan phosphate [29]); *Sclerotinia sclerotiorum *(SSG [30]); and *Phellinus linteus *(powder [31] and aqueous, alcohol-precipitated extract [32]). A furanose extract from *P. quiquefolium *and pectins from *Buplerum falcatum *and *Malus *(apple) spp. have also been shown to enhance immune function in healthy young animals [33-35]. *Cyamopsis tetragonolobus *galactomannan (guar gum) or highly methoxylated pectin feeding exerted numerous stimulating effects on antibody production in older animals [36].

Evidence for the effectiveness of oral polysaccharides against infection and immune challenges has been mainly demonstrated in animals. Immune stimulating effects have been shown in resting and exercise-stressed animals with thioglycollate, clodronate, or HSV-1 injections fed *Avena *(oat) spp. soluble glucans [37-41]; animals injected with or fed *E. vermiformis *and fed *Avena *spp. particulate glucans [42,43]; animals with *E. coli *injections fed *L. digitata *glucans (laminarin) [44]; animals with HSV injections fed *U. pinnatifida *fucoidans [45]; animals with *Staphylococcus aureus *or *Candida albicans *injections fed *S. cerevisiae *glucans (scleroglucan) [29]; and animals with fecal solution injections fed an aqueous extract of *A. subrufescens *(*A. blazei *Murrill) [46].

Additional controlled human and animal studies have shown anti-inflammatory and anti-allergy effects of some polysaccharide products. In an RCT of adults with seasonal allergic rhinitis, *S. cerevisiae *β-1,3;1-6 glucans decreased IL-4, IL-5 and percent eosinophils, and increased IL-12 in nasal fluid [47], while a placebo-controlled study of patients with recurrent aphthous stomatitis (canker sores) consuming β-1,3;1-6 glucans found increased lymphocyte proliferation and decreased Ulcer Severity Scores [48].

Animal models of inflammatory bowel disease have shown anti-inflammatory effects of *Cladosiphon okamuranus *Tokida fucoidans [49], *Cyamopsis tetragonolobus *galactomannans [50], *Malus *spp. pectins [51], and mixed polysaccharide supplements [52]. Animals challenged with ovalbumin have demonstrated anti-inflammatory/allergy effects of *A. subrufescens *aqueous extracts [22], an aqueous extract *of Ganoderma tsugae *[53], and *Pyrus pyrifolia *pectins [54]. Anti-inflammatory effects have also been seen in animals with cotton pellet implantations fed a *Pholiota nameko *heteroglycan (PNPS-1) [55].

*Trametes versicolor *glucans have demonstrated anti-cancer effects in humans. In two RCTs and five controlled trials, PSK from *T. versicolor *mycelia increased survival of advanced stage gastric, colon and colorectal cancer patients [56-62] with one study showing increased immune parameters (including blood NK cell activity, leukocyte cytotoxicity, proportion of helper cells and lymphocyte suppressor cells) [62]. An RCT of advanced stage lung cancer patients consuming PSP from *T. versicolor *fruit bodies found increased IgG and IgM antibodies and total leukocyte and neutrophil counts, along with a decrease in the number of patients withdrawing from the study due to disease progression [63]. An RCT of ovarian or endometrial cancer patients consuming *A. subrufescens *glucans showed increased NK cell activity and fewer chemotherapy side effects [64].

In numerous animal models of cancer, a wide range of polysaccharides have shown anti-tumorogenic effects. Glucan products sourced from *A. subrufescens *demonstrating anti-cancer activities in animal models include an aqueous extract [65], an aqueous, acid-treated extract [66], and an aqueous extract with standardized levels of β-glucans [23]. Anti-cancer effects have been reported following intake of aqueous extracts of *G. lucidum *[67-69]; the powder and D fraction of *G. frondosa *[70-72]; *Hordeum vulgare *β-glucans [73-76]; *Laminaria angustata *powder [77]; *Lentinula edodes products *(powders [70,78,79], SME [80], β-glucans [27], and lentinan [81,82]); *Pleurotus ostreatus *powder [70], *Saccharomyces cerevisiae *particulate β-1,3;1,6 and β-1,3glucans[27,73]; and a glucan from *Sclerotinia sclerotiorum *(SSG) [30,83]. A glucomannan from *L. edodes *(KS-2) improved survival of animals with cancer cell injections [84]; apple and citrus pectins have exerted anti-cancer effects, including decreased tumor incidence [85-90]. Finally, heteroglycans from *Lycium barbarum *(LBP_3p_), *Lentinus lepidus *(PG101) and A. *subrufescens *(ATOM) demonstrated a number of immune stimulating effects in animal cancer models [91-93]. Interestingly, only one animal study has been performed using glucans from *T. versicolor *(PSP): animals with cancer cell implantations showed decreased tumor growth and vascular density [94].

Most polysaccharide products appear to be safe, based on NOAEL, acute and/or chronic toxicity testing in rodents (Table 6). As would be expected, powders, extracts and products that have not been fully characterized pose the most concerns. Other than for aloe vera gel, which was shown in a small human trial to increase the plasma bioavailability of vitamins C and E [95], the impact of polysaccharide intake on the absorption of nutrients and medications is not known. While one rat toxicity study raised concerns when guar gum comprised 15% of the daily diet [96], the product was safe in humans studies when 18-39.6 g/day was consumed for up to a year (Table 4). Product contamination may explain three case reports of hepatotoxicity and/or death following intake of an *A. subrufescens *aqueous extract [97]. Seven animal studies reporting positive immunologic effects of *A. subrufescens *extracts in healthy animals or animals with cancers found no evidence of toxicity (Tables 1 and 2). In humans, six weeks of *A. subrufescens *glucans intake was safe for cancer patients, and four months of 3 g/day intake by 24 healthy adults and 24 adults with liver disease reported no evidence of toxicity (Table 4). Another case report associated liver toxicity with *G. lucidum *intake, but the elderly subject also took an unidentified product a month previous to her admission for testing [98]. Three animal studies reported immunologic benefits and no adverse effects following intake of *G. lucidum *aqueous extracts; in one study intake was 5% of the diet for 5 months (Table 1). While adverse effects were also reported in a study in which 10 adults consumed 4 g/day *L. edodes *powder for 10 weeks [99], immunologic animal studies reported no ill effects of either *L. edodes *powder (5 studies, up to 5% of the diet up to nine months) or extract (7 studies, up to 40 days intake) (Tables 1 and 3). Finally, while intake of 319 mg/kg of an aqueous extract of *P. ostreatus *by mice for 1 month caused hemorrhages in multiple tissues [100], there was no reported toxicity when mice consumed the mushroom powder as 5% of their diet for nine months (Table 3). While ≥1 gram/day of *T. versicolor *glucan products were safely consumed by cancer patients for up to 10 years, the long-term effects of ingestion of the other polysaccharide products discussed in this review is also not known.

## Discussion

The majority of studies that qualified for inclusion in this review employed models investigating immune stimulation; fewer explored anti-inflammatory effects. Animal studies reported immune system effects in the gut, spleen, bone marrow, liver, blood, thymus, lungs, and saliva; controlled human studies reported evidence of immune stimulation in the blood, anti-inflammatory effects in nasal lavage fluid and improved survival in cancer patients. The literature is highly heterogenous and is not sufficient to support broad structure/function generalizations. For the limited number of studies that investigated well-characterized, isolated products (primarily glucan products), effects can be unequivocally attributed to polysaccharides. Such associations are certainly more tenuous when considering product powders or products obtained by extraction methods designed to isolate polysaccharides, but without complete compositional analyses.

Dietary polysaccharides are known to impact gut microbial ecology [101,102], and advances in microbial ecology, immunology and metabolomics indicate that gut microbiota can impact host nutrition, immune modulation, resistance to pathogens, intestinal epithelial development and activity, and energy metabolism [103-107]. Other than fucoidans, the polysaccharides discussed in this review appear to be at least partially degraded by bacterial enzymes in the human digestive tract (Table 7). Arabinogalactans, galactomannans, a glucan (laminarin), glucomannans, and mixed polysaccharide products (Ambrotose^® ^products) have been shown to be metabolized by human colonic bacteria. Orally ingested fucoidans, glucans and mannans (or their fragments) have been detected in numerous tissues and organs throughout the body [73,108,109], (Carrington Laboratories, personal communication). We know of no study that has determined the specific identity of orally-ingested polysaccharide end products in animal or human tissues.

**Table 7:** Fate of Immunomodulatory Polysaccharide Products Following Oral Intake

Category	Product	Metabol-ized by human gut bacteria?	Study type	Fate (method: tissues detected)	References
Arabinogalactans	*Larix *spp.	yes	*in vitro*	NA	[163-169]

Fucoidans	*Undaria pinnatifida*	no	*in vitro*	Ab: human plasma	[108,170]

Galactomannans	*Cyamopsis tetragonolobus *(partially hydrolyzed guar gum)	yes	*in vivo*	NA	[171]
	
	*Cyamopsis tetragonolobus *(guar gum)	yes	*in vitro*	NA	[167]

Glucans	*Hordeum vulgare*	NA	*in vivo*	Fluorescein-labeled: mouse Mø in the spleen, bone marrow, lymph nodes	[73]
	
	*Laminaria digitata *(laminarin)	yes	*in vitro*	NA	[29,170,172]
	
	*Sclerotium rofsii *(scleroglucan) glucan phosphate, *Laminaria *spp. (laminarin)	NA	*in vivo*	Alexa Fluor 488-labeled: mouse intestinal epithelial cells, plasma, GALT	[29]
	
	*Saccharomyces cervisiae *(particulate)	NA	*in vivo*	Fluorescein-labeled: mouse macrophage in the spleen, bone marrow, lymph nodes	[73]
	
	*Trametes versicolor* (PSK)	NA	*in vivo*	^14^C-labeled: rat and rabbit serum; mouse GI tract, bone marrow, salivary glands, liver, brain, spleen, pancreas	[173]

Mannans	*Aloe barbadensis *(aloemannan)	yes	*in vitro*	FITC-labeled: mouse, GI tract	[121,174]
	
	*Aloe barbadensis* (gel powder)	yes	*in vitro*	NA	[163]
	
	*Aloe barbadensis *(acemannan)	NA	*in vivo*	^14^C-labeled: dog systemic, particularly liver, bone marrow, gut, kidney, thymus, spleen	(Carrington Laboratories, personal communication)

Mixed polysaccharide products	Ambrotose complex^®^, Advanced Ambrotose^® ^powder	yes	*in vitro*	NA	[163,175]

Pectins	NA	yes	*in vitro*	NA	[165-167,176]
	
	*Buplerum falcatum *(bupleuran 2IIc)	NA	*in vivo*	Ab bound: mouse Peyer's patch, liver	[109]

One can only speculate upon the mechanisms by which the polysaccharides discussed in this review influence immunologic function, particularly when one considers the exceedingly complex environment of the GI tract. It is possible that fragments of polysaccharides partially hydrolyzed by gut bacteria may either bind to gut epithelia and exert localized and/or systemic immune system effects, or be absorbed into the bloodstream, with the potential to exert systemic effects. Current studies investigating the link between the bioconversion of dietary polysaccharides, their bioavailability and their downstream effects on the host metabolism and physiology are utilizing metabolomic and metagenomic approaches that can detect and track diverse microbial metabolites from immunomodulatory polysaccharides [103]. These and other innovative approaches in the field of colonic fermentation are providing novel insights into gut microbial-human mutualism [110,111], its impact on regulating human health and disease, and the importance of dietary modulation [112-115].

Additional RCTs of well-characterized products are needed to more completely understand the immunomodulatory effects and specific applications of oral polysaccharides. Such studies will need to better investigate the optimal timing and duration for polysaccharide ingestion. That is, should they be consumed continuously, before, at the time of, or after exposure to a pathogen or environmental insult? Only a few studies have actually investigated the impact of timing of polysaccharide intake to achieve optimal benefits. Daily feeding with some polysaccharides appears to result in tolerance (and diminished benefits); this has been demonstrated for some mushroom β-glucans [3,26]. For those polysaccharides whose immunologic effects are dependent on their prebiotic activities, regular feeding would be presumed necessary.

## Conclusions

The dietary polysaccharides included in this review have been shown to elicit diverse immunomodulatory effects in animal tissues, including the blood, GI tract, and spleen. In controlled human trials, polysaccharide intake stimulated the immune system in the blood of healthy adults, dampened the allergic response to a respiratory inflammatory agent, and improved survival in cancer patients. Additional RCTs of well-characterized products are needed to more completely understand the immunomodulatory effects and specific applications of oral polysaccharides

## List of abbreviations

♀: female; ♂: male; Ab: antibody; AIDS: autoimmune deficiency syndrome; AOM: azoxymethane; BBN: N-butyl-N'-butanolnitrosamine; BLCL: Burkitt's Lymphoma Cell Line; BW: body weight; CBC: complete blood count; CD: cluster of differentiation; CFU: colony forming unit; ConA: concanavalin A; CXCR: CXC chemokine receptor; DMBA: 7,12-dimethylbenz*(a)*anthracene; DMH: N-N'-dimethylhydrazine; DMN: dimethylhydrazine; DSS: dextran sulfate sodium; EBV: Epstein-Barr virus; GALT: gut-associated lymphoid tissue; GI: gastrointestinal; H_2_0_2: _hydrogen peroxide; HSV: herpes simplex virus; ICR: imprinting control region; ID: intradermal; IEL: intraepithelial lymphocytes; IFN-λ: interferon gamma; IG: intragastric; IgA: immunoglobulin A; IgE: immunoglobulin E; IgG: immunoglobulin G; IgM: immunoglobulin M; IL: interleukin; IMC: invasive micropapillary carcinoma; IN: intranasally; IP: intraperitoneal; IV: intravenous; LPS: lipopolysaccharide; Mø: macrophage; mAb: monoclonal antibody; 3-MCA: methylcholanthrene; MLN: mesenteric lymph nodes; MM-46 carcinoma: mouse mammary carcinoma; MW: molecular weight; NK: natural killer; NOAEL: no observable adverse effect level; OVA: ovalbumin; PBL: peripheral blood leukocytes; PBMC: peripheral blood mononuclear cells; PHA: phytohaemagglutinin; PMA: phorbol 12-myristate 13-acetate; PML: polymorphonuclear lymphocyte; RCT: randomized, controlled trial; RNA: ribonucleic acid; SC: subcutaneous; SD rats: Sprague Dawley; TCR: T cell receptor; TLR: toll like receptor; TNF-α: tumor necrosis factor alpha; UC: ulcerative colitis; WT: wild type.

## Competing interests

The authors are employees of the Research & Development Department at Mannatech, Incorporated, which sells two of the polysaccharide products (Ambrotose^® ^powder and Advanced Ambrotose^® ^powder) discussed in this review.

## Authors' contributions

JER and EDN conducted literature searches and wrote the manuscript. RAS provided technical guidance. All authors read and approved the final manuscript.
